# Poor Housing Quality and Mental Health in Public Housing: Age Differences and Neighborhood Contexts

**DOI:** 10.1093/geroni/igaf122.2119

**Published:** 2025-12-31

**Authors:** Zhirui Chen, Yilin Wang, Samantha Teixeira, Rebekah Coley

**Affiliations:** Boston College, Framingham, Massachusetts, United States; Boston College, Chestnut Hill, Massachusetts, United States; Boston College School of Social Work, Chestnut Hill, Massachusetts, United States; Boston College, Chestnut Hill, Massachusetts, United States

## Abstract

Poor housing quality presents a significant challenge for public housing residents in the U.S., which impedes their health and wellbeing. This study examined the association between housing quality and mental health among public housing residents and how age and neighborhood contexts moderated this relationship. Data come from the first wave of a mixed-methods evaluation of public housing redevelopment, the Housing Opportunity and Mobility Experiment (HOME), located in a large public housing community. The analytic sample included 448 adults (ages 18-97), with data collected through in-person and online surveys between October 2022 and January 2024. Results of multiple linear regression showed that poor housing quality (e.g., mold, pests) was associated with increased symptoms of anxiety, depression, and perceived stress among public housing residents. Older adults who experienced lower housing quality reported worse mental health relative to their younger counterparts. Additionally, neighborhood problems, as a risk factor, was significantly associated with worse mental health outcomes and further exacerbated the links between housing quality and mental health more for older adults than for younger populations. These findings have implications for the development of age-responsive mental health interventions tailored to the unique contexts of public housing communities. As many public housing developments are undergoing redevelopment, often with the goals of enhancing supportive services, there is an opportunity to incorporate targeted mental health services, particularly for older residents who are more vulnerable to environmental stressors. To better support residents’ mental health, initiatives are needed to address community stressors such as drug presence and violence.

